# A case series of *Candidozyma auris* in Viet Nam

**DOI:** 10.1186/s12879-026-12690-3

**Published:** 2026-02-02

**Authors:** Van Phuc Pham, Nam Xuan Ha, Huan Thi Nguyen, Le Nguyen Minh Hoa, Dinh Phu Vu, Van Dinh Trang, H Rogier van Doorn, Pham Ngoc Thach

**Affiliations:** 1 VNU University of Medicine and Pharmacy, Ha Noi, Viet Nam; 2 National Hospital for Tropical Diseases, Ha Noi, Viet Nam; 3 Oxford University Clinical Research Unit (OUCRU), Ha Noi, Viet Nam; 4 Centre for Tropical Medicine and Global Health, Nuffield Department of Medicine, University of Oxford, Oxford, UK

**Keywords:** *Candidozyma auris*, *Candidaauris*, Mycosis, Fungal infection, Hanoi, Viet Nam

## Abstract

*Candidozyma auris* is an emerging health threat, especially in healthcare settings due to its resistance to several classes of antifungals leading to high mortality. In Viet Nam, the burden of this fungal pathogen is unknown because of little available data. In this report, six cases with *C. auris* isolation between 2023 and 2024 at the National Hospital for Tropical Diseases, a tertiary referral hospital in northern Viet Nam, are described. Our findings highlight the first report of *C. auris* in the north of Viet Nam and underscore the importance of prevalence surveys in the future.

## Introduction

*Candidozyma auris (*formerly *Candida auris)* is an emerging fungal pathogen linked with healthcare-associated infections. Since the first report in 2009 in Japan, cases of nosocomial *C. auris* infections have been reported in over forty countries in six continents by 2020 [1, 2]. *C. auris* is notorious for outbreaks in healthcare facilities and high mortality due to resistance to multiple classes of antifungal agents [3, 4]. Although *C. auris* does not present as a frequent clinical problem yet in Viet Nam, the actual burden of *C. auris* is unknown due to little available data. Three case studies describing ten cases of *C. auris* infection in Viet Nam have been published d from the south of the country [5–7]. Here, we report six patients of isolation of *C. auris* from infected and colonized patients at the National Hospital for Tropical Diseases (NHTD), a 500-bed tertiary referral hospital for infectious diseases in northern Viet Nam. To our knowledge, these are the first cases described from the north of Viet Nam.

## Case presentation

In this case series, six adult patients with *C. auris* at the Intensive Care Unit (ICU) between November 2023 and June 2024 are described, in which three were diagnosed as infection and three were colonization. Infections were defined as clinical deterioration at the time of C. auris isolation, despite antibiotic treatment, responding well to antifungal therapy. Cases where C. auris was isolated without clinical manifestations and that improved without antifungal treatment were defined as colonization. In total, *C. auris* was isolated 10 times, including from six endotracheal aspirate (ETA), from three central venous catheter (CVC), and once from skin. No isolate was from blood. Species identification was done using the MALDI-TOF MS (bioMérieux, Marcy l’Étoile, France). Antifungal susceptibility testing was done using the broth microdilution method [8], and the interpretation was according to European Committee on Antimicrobial Susceptibility Testing (EUCAST) 2025 and Clinical and Laboratory Standards Institute (CLSI) 2022 guidelines [8, 9]. The susceptibility of four isolates is shown in Tables 1 and 2.

**Table 1:** Main clinical characteristics of six patients with *Candidozyma auris* in Northern Viet Nam

Characteristics	Case 1	Case 2	Case 3	Case 4	Case 5	Case 6
Age	65	38	73	46	64	53
Sex	Male	Male	Male	Male	Female	Male
Medical history	Chronic renal failure Hypertension Long-term use of steroids	Untreated hepatitis B	Rheumatoid arthritis Hypertension	None	Hypertension	Alcoholic cirrhosis
Diagnosis on admission	Surgical site infection at left tibia	Acute hepatic failure	CAP	Meningitis	CAP	CAP
Length of admission before transfer	63 days	3 days	123 days	4 days	1 day	22 days
Diagnosis after transfer to NHTD	Surgical site infection VAP Septic shock	Hepatic encephalopathy VAP Septic shock	VAP Septic shock	Meningitis Viral encephalitis VAP UTI	VAP Septic shock	VAP Septic shock
Specimens for *C. auris* (No. of isolates)	CVC (1) ^a^, ETA (1)	ETA (1)	CVC (1) ^a^, skin (1)	ETA (2)	ETA (2)	CVC (1)
Time from admission to first isolation	6 days	14 days	50 days	50 days	13 days	52 days
Time from taking sample to isolation	3 days for both	2 days	2 day for catheter, 7 days for skin	3 days for first isolate, 4 days for second one	3 days for first isolate, 4 days for second one	2 days
Time to culture negative ^b^	16 days	6 days	Not done	16 days	22 days	Not done
Deterioration at time of isolation	Yes	No	Yes	No	No	Yes
Antifungal therapy	Yes	No	Yes	Yes	No	Yes
Prior to isolation (days)	No	No	Fluconazole (10) Caspofungin (47)	Caspofungin (14)	No	No
After isolation (days)	Caspofungin (7) Anidulafungin (14)	No	Caspofungin (18)	No	No	Caspofungin (6)
Other potentially pathogenic organisms isolated	*A. baumannii* *K. pneumoniae* *S. maltophilia*	*K. pneumoniae*	*P. aeruginosa*	*A. baumannii* *P. aeruginosa* *C. albicans*	*S. maltophilia*	*A. baumannii* *P. aeruginosa*
Length of admission	106 days	21 days	91 days	90 days	50 days	58 days
Outcome at discharge	Recovered	Poor prognosis	Poor prognosis	Died	Recovered	Poor prognosis
Invasive procedures at time of first isolation of *C. auris*						
Mechanical ventilation	Yes	Yes	Yes	Yes	Yes	Yes
Central venous catheter	Yes	Yes	Yes	Yes	Yes	Yes
Arterial line	Yes	Yes	Yes	Yes	Yes	Yes
CRRT	Yes	Yes	Yes	Yes	No	Yes
Hemodialysis	Yes	No	No	No	No	No
Parenteral nutrition	Yes	Yes	Yes	Yes	Yes	No
Plasma exchange	No	Yes	No	Yes	No	No

Note: CAP, Community-acquired pneumonia; CRRT, Continuous renal replacement therapy; ETA, Endotracheal aspiration; NHTD, National Hospital for Tropical Diseases; UTI, Urinary tract infections; VAP, Ventilator-acquired pneumonia

^a^ indicates the first isolation of *C. auris*

^b^ duration from the first isolation of *C. auris* to fungal culture negative

**Table 2:** Antifungal susceptibility testing of *Candidozyma auris*

Case	Azoles		Echinocandins				Polyenes
	Fluconazole	Voriconazole	Anidulafungin	Caspofungin	Micafungin	Amphotericin B deoxycholate	
Case 1	> 256.00 (R)	> 8.00	0.25 (S)	> 8.00	0.25 (S)	2.00 (R)	
Case 2	-	-	-	-	-	-	
Case 3	64.00 (R)	0.12	0.12 (S)	0.25	0.12 (S)	1.00 (S)	
Case 4	> 128.00 (R)	-	0.25 (S)	> 8.00	0.50 (R)	0.50 (S)	
Case 5	-	-	-	-	-	-	
Case 6	128.00 (R)	-	0.12 (S)	> 8.00	0.12 (S)	2.00 (R)	

Note: Data were presented with minimum inhibitory concentration (MIC) value and susceptibility whose interpretation according to the US (CLSI) and EUCAST guidelines. The susceptibility testing was performed on the first isolate of *C. auris* [8, 9]

R, Resistant; S, Susceptible; -, Not done

### Case 1 65M November 2023 – *C. auris* from CVC and ETA

The patient was admitted to a hospital in Ha Noi after a road traffic accident and had external fixation for a left tibia fracture. His medical history included stroke, hypertension, and chronic renal failure with hemodialysis since 2021. After surgery, the patient was transferred to Phu Tho provincial for 1 month, but his condition became worsened with fever and pus from the wound. He was transferred to another hospital in Ha Noi and diagnosed with surgical site infection caused by *Klebsiella pneumoniae* and treated with antibiotics (meropenem and colistin), drainage, and steroids. After an additional he was transferred to NHTD. He presented with fever, elevated inflammatory biomarkers (white blood cell counts [WBC] 10.4 10^9^/L, C-reactive protein [CRP] 84.3 mg/L, and procalcitonin [PCT] 29.1 ng/mL), chest X-ray showing opacities in the left lung, a fractured rib and drains in situ (tibia). A diagnosis of hospital-acquired pneumonia (HAP) and suspicion of sepsis were established. Antibiotics including doripenem and daptomycin were given, but no supplemental oxygen therapy was needed. On day 5, *Acinetobacter baumannii* was isolated from blood, and antibiotics were switched to ampicillin- sulbactam, colistin, and daptomycin. On day 6, *C. auris* was first isolated from the tip of a removed CVC originating from the referring healthcare facility, and caspofungin was initiated due to persistent fever and elevated biomarkers. Because the antifungal susceptibility testing showed MIC > 8 mg/L (upper limit of the assay) to caspofungin, antifungals were switched to anidulafungin for 14 days, which had more favourable MIC values. On day 15, the patient developed severe dyspnea, hypotension, and required mechanical ventilation with vasopressors. He was diagnosed with VAP and *K. pneumoniae* was isolated from ETA, for which he received treatment with meropenem and colistin. Repeat cultures of CVC for *C. auris* remained positive on day 11, then turned to negative on day 21. Following this, he experienced multiple episodes of VAP and was under continuous antibiotic treatment. At day 51, culture yielded a second isolate of *C. auris* from ETA in the presence of fever and elevated inflammatory biomarkers (WBC 16.9 10^9^/L, CRP 124.6 mg/L, and PCT 1.0 ng/mL) and under treatment with antibiotics (ampicillin-sulbactam, ceftazidime, daptomycin, and colistin). Anidulafungin was re-started for 7 days. Subsequent fungal cultures taken 9 days later remained negative. On day 64, the patient suffered from VAP caused by *Stenotrophomonas maltophilia*. On day 83, the patient’s condition had improved and antibiotic therapy was discontinued. At day 106, his leg was stable without drainage or swelling, and he was transferred to a lower-level hospital while still requiring mechanical ventilation.

### Case 2 38M March 2024 – *C. auris* colonization of airways

The patient was admitted to Vinh Phuc provincial hospital for three days due to fatigue, jaundice, and then transferred to NHTD. An initial diagnosis of acute hepatic failure due to hepatitis B virus infection was made, and treatment was started with entecavir. After four days, the patient had decreased level of consciousness, upper abdominal pain, oliguria, and an increased PCT value (0.73 ng/ml). He was diagnosed with hepatic encephalopathy (ALT 1124 U/L, AST 413 U/L, total bilirubin 534.9 umol/L, direct bilirubin 370 umol/L, prothrombin time 41.2 s, INR 3.64, and serum ammonia 87.4 umol/L) and suspicion of bacterial bloodstream infection. The patient was intubated and plasma exchange and antibiotic treatment (ertapenem) were started. On day 14 antibiotic therapy was discontinued and *C. auris* and *K. pneumoniae* were isolated from ETA. Antifungal therapy and antibiotics were not given due to the patients improving clinical condition. After 6 days, a follow-up ETA culture remained negative for yeast but still grew *K. pneumoniae* while the patient had again deteriorated. Antibiotics (meropenem and colistin) were re-initiated for a diagnosis of VAP. On day 21, his legal representative requested for the patient to be discharged to die at home. His hepatic encephalopathy had not improved and he was not breathing by himself.

### Case 3 73M February 2024 – *C. auris* from CVC and skin

The patient with a medical history of rheumatoid arthritis with long-term use of corticosteroids and hypertension presented at NHTD. He had been discharged 4 days ago where he had been admitted since October 2023 with severe community-acquired pneumonia (CAP), complicated by multiple episodes of VAP due to multidrug-resistant bacteria (*A. baumannii*,* K. pneumoniae*, and *Pseudomonas aeruginosa* isolated from ETA) and invasive fungal infections (*C. albicans and C. parapsilosis* isolated from ETA), and septic shock. The management included intubation, vasopressors, antibiotics, and antifungal therapy (oral fluconazole and caspofungin). After 123 days, he was discharged for home care at the request of his legal representatives despite being on treatment for septic shock, requiring mechanical ventilation, and persistent high levels of inflammatory biomarkers (WBC 23.1 10^9^/L, CRP 51.9 mg/L, and PCT 0.17 ng/mL). The patient was thus re-admitted after four days and was put back on ventilator, vasopressors, and antibiotics (meropenem, colistin, and vancomycin). Chest X-ray showed bilateral heterogeneous opacities. On day 20 of the second admission, *P. aeruginosa* was cultured from ETA and the patient again developed multiple episodes of VAP. On day 51, while treated with meropenem and fosfomycin, *C. auris* was first isolated from the tip of a removed CVC. Catheters were changed and an 18-day course of caspofungin was started because of clinical deterioration including a new episode of fever, hypotension, and elevated inflammatory biomarkers (WBC 17.9 10^9^/L, CRP 27.0 mg/L, and PCT 0.17 ng/mL). On day 70, *C. auris* was isolated from skin lesions but antifungal treatment was not re-introduced. Follow-up fungal cultures from CVC were not done, but *C. auris* was still isolated from skin on day 80. On day 91, the patient’s relative again requested discharge because of his poor response. At discharge, he was still requiring ventilation and had elevated biomarkers (WBC 12.4 10^9^/L, CRP 155 mg/L, and PCR 1.87 ng/mL).

### Case 4 46M May 2024 – *C. auris* colonization in ETA

The patient without underlying disease was hospitalized in Bac Giang provincial hospital after one day of fever, severe headache, and nausea. He was diagnosed with meningitis and managed with ceftriaxone and dexamethasone. After four days, he developed a seizure and deteriorating consciousness, and required intubation and was transferred to NHTD. A diagnosis of bacterial meningitis and suspicion of encephalitis were made, and ceftriaxone was continued with addition of 30-day acyclovir. The cerebral magnetic resonance imaging (MRI) showed lesions compatible with *Herpes simplex virus* (HSV) infection at the putamen, insula, and temporal lobes. Cerebrospinal fluid (CSF) analysis on day 1 was suspicious for viral etiology (white cell counts 27 cells/mm^3^, glucose 5.74 mmol/L, and protein 0.47 g/L), but no pathogen in CSF was detected by culture, Xpert MTB/ RIF, HSV PCR, or JEV PCR. On day 7 after admission to NHTD, *C. albicans* was isolated from ETA and the patient was treated with 14 days of caspofungin. On day 17, autoimmune encephalitis was suspected due to poor recovery after treatment of meningitis and encephalitis, and an anti-N-methyl-D-aspartate receptor (anti-NMDAR) test in CSF was requested. Despite a negative result, the patient received plasma exchange and 14-day rituximab for autoimmune encephalitis. On days 50 and 79, *C. auris* was isolated from ETA but left untreated in the absence of clinical VAP/deterioration and inflammatory biomarkers elevation. Follow-up cultures on days 65 and 88 remained negative. During hospitalization, the patient suffered from multiple episodes of nosocomial infections, including VAP due to *A. baumannii* and catheter-associated urinary tract infection (CAUTI) with *P. aeruginosa*. After many courses of antibiotics including meropenem, linezolid, colistin, long-term corticoids, anticonvulsants, CRRT, and plasma exchange, his symptoms were getting better except for persistent convulsions. Although an improvement in the latest cerebral MRI was observed, the patient remained comatose and ventilator dependent. On day 90, he died of sudden cardiac arrest.

### Case 5 64F June 2024 – *C. auris* colonization in ETA

The patient with a history of hypertension presented at Thai Binh provincial hospital with one day of fever and dyspnea, and was diagnosed with CAP. A chest CT scan showed consolidation across both lungs. After one day, symptoms deteriorated, and the patient was intubated and transferred to NHTD. A diagnosis of pneumonia with septic shock was made, with hypotension and high levels of inflammatory biomarkers (WBC 12.4 10^9^/L, CRP 241.9 mg/L, and PCT 2.17 ng/mL). The management consisted of vasopressors and broad-spectrum antibiotics (imipenem and vancomycin). On day 29, *S. maltophilia* was isolated from ETA and a diagnosis of VAP was established. On day 13, *C. auris* was first isolated from ETA, and again on day 29. In both instances, the follow-up cultures 5 days later remained negative. In the absence of exacerbation and good response with antibiotic therapy, no antifungal medication was initiated. On day 50, the patient, while still ventilator dependent, was transferred back to the provincial hospital.

### Case 6 53F June 2024 – *C. auris* on CVC

The patient with a history of alcoholic liver cirrhosis was admitted at Bac Giang provincial hospital after two days of fever, severe cough, and dyspnea. He was diagnosed as having CAP. On admission, he was intubated and developed VAP on day 5 with isolation of *A. baumannii* from ETA. The management included antibiotics (meropenem, colistin) and CRRT. After two weeks, his condition worsened with persistent fever and low blood pressure. The patient was diagnosed with septic shock and transferred to NHTD. On day 1, ETA culture was positive for *A. baumannii* in addition to infiltration and opacities in the chest X-ray compatible with VAP. Broad-spectrum antibiotics comprising meropenem, colistin, ampicillin-sulbactam, and linezolid was initiated. *P. aeruginosa* was also isolated from ETA five days later. The patient developed multiple episodes of VAP with this pathogen and suffered from multiorgan dysfunction. On day 52, while he was on ventilator and antibiotic therapy (meropenem, colistin, and fosfomycin), *C. auris* was isolated from the tip of a removed CVC specimen accompanied by a new episode of fever, impairment of respiratory function, and elevated inflammatory biomarkers (WBC 8.0 10^9^/L, CRP 40.3 mg/L, and PCT 0.54 ng/mL). Caspofungin was prescribed, and the catheter was changed. However, he did not response well with regimens (WBC 13.8 10^9^/L, CRP 285.5 mg/L, and PCT 6.59 ng/L). Six days later, his legal representative asked to discharge him to die at home.

## Discussion

In Viet Nam, the first report of *C. auris* was published in 2020 with two subsequent reports in 2024, describing a total of ten cases from the south. Our study represents the first record of colonization and infection with *C. auris* from the north of Viet Nam, confirming its presence throughout the country. Nevertheless, those cases were found through routine diagnostics based on clinical symptoms. IPC screening for carriage was not performed. In general, demographic and clinical characteristics of patients infected by *C. auris* in our study are similar to the other reports from Viet Nam [5–7]. All cases were critically ill at the time of isolation and had previously been exposed to ICU admission, mechanical ventilation, broad-spectrum antibiotics, and multiple invasive medical procedures. Those risk factors for *C. auris* in six cases were consistent with those in recent reviews [3, 4]. Although culture from blood and ETA/CVC was conducted as routine diagnostics on admission to ICU, in the absence of full admission screening for carriage, pre-existing carriage or local acquisition during stay in our unit could not be assessed, except case 1 whose first isolate was from the referring healthcare facility.

Three of six cases were diagnosed with invasive infection, of whom one recovered. Clinical management consisted of catheter replacement and prompt echinocandin therapy (case 1, 3, and 6). Colonization with *C. auris* was identified in the other three individuals without the introduction of antifungal agents. The high mortality and poor prognosis at discharge among cases in the present study are likely secondary to the critical clinical status. Moreover, as a marker of treatment response or discontinuation of contact isolation, negative results (time to culture negative) in our study did not indicate microbiologic clearance. Antifungal susceptibility testing was conducted for 4/6 isolates (due to supply issues with Sensititre plates). Compared to previous studies in Viet Nam, our isolates were highly resistant to fluconazole (four isolates) (Table 2) [5–7]. However, we currently do not have access to molecular typing or the whole genome sequencing, and no molecular epidemiology analysis was undertaken.

Because of the association with outbreaks in healthcare settings, after *C. auris* was isolated, patients were placed in contact isolation within a single room and nursed separately using gowns, gloves, and masks as PPE, as per hospital protocols. Transferring hospitals were informed. CVC or endotracheal tube were replaced if present. Environmental cleaning and disinfection were conducted using hydrogen peroxide. A refresher training on precautions for *C. auris* was mandatory for all healthcare personnel in ICU. Patient and environmental screening are not part of current *C. auris* protocols at our hospital. Table 3 depicts the chronological order of reports in Viet Nam [5–7]. All patients required prolonged ICU admission except for one case. In 2020, the first case report of *C. auris* colonization whose specimens originated from Viet Nam was isolated in Australia [5]. Two case series were totally reported in Viet Nam, in which one identified *C. auris* only by RT-PCR in blood [7] and one simultaneously tested both VITEK2 Compact and MALDI-TOF MS techniques [6]. AST presented the resistance varied; of note, resistance to amphotericin B was observed in six isolates. Furthermore, only one study used molecular typing and reported clade I (Table 3) [7]. Only five cases recovered, which emphasized high mortality among patients having isolation of *C. auris* in the resource-limited setting. To date, an official guideline for managing *C. auris* colonization or infection has not yet been issued in Viet Nam, posing a challenge in diagnosis and treatment for physicians. Further studies are needed to determine the prevalence, resistance, and burden of *C. auris* to facilitate the management.

**Table 3:** Chronological order of reports of *Candidozyma auris* in Viet Nam

Publication	Region (Sample collection period)	Hospital (Department of isolates)	No. of patients (No. of isolates)	Specimen (No. of isolates)	Clade	Method of identification	Antifungal resistance (No. of isolates)	Outcome at discharge (No. of patients)
Ouli/2020/Australia [5]	South (2019)	N/A	1 (1)	IPC screening samples (1)	N/A	Culture (MALDI-TOF MS) and genome sequencing	Fluconazole (1) Amphotericin B (1)	Poor prognosis (1)
Thong/2024/Viet Nam [6]	South (2024)	Cho Ray (Department of pulmonary medicine and ICU)	4 (5)	Blood (2) Sputum (1) Stool (1) Urine (1)	N/A	Culture (VITEK^®^ 2 Compact and MALDI-TOF MS)	Amphotericin B (1)	Recovered (2) Died (2)
Hong/2024/Viet Nam [7]	South (2024)	N/A (ICU)	5 (5)	Blood (5)	I	RT-PCR	Amphotericin B (4) Caspofungin (1)	Recovered (1) Poor prognosis (2) Died (2)
This report	North (2024)	NHTD (ICU)	6 (10)	CVC (3) ETA (6) Skin (1)	N/A	Culture (MALDI-TOF MS)	Fluconazole (4) Amphotericin B deoxycholate (2) Micafungin (1)	Recovered (2) Poor prognosis (3) Died (1)

Note: CVC, Central venous catheter; ETA, Endotracheal aspirate; ICU, Intensive care unit; IPC, Infection prevention and control; MALDI-TOF MS, Matrix assisted laser desorption/ionization time-of-flight mass spectrometry; NHTD, National Hospital for Tropical Diseases; RT-PCR, Real-time polymerase chain reaction

## Conclusions

We describe the isolation of *C. auris* in ICU setting and confirm presence of *C. auris* in the north of Viet Nam. Comparison with earlier reports from the south of Viet Nam shows considerable variation in the resistance to antifungal agents and a high mortality.

## Data Availability

Please contact the corresponding author or Van Phuc Pham, M.D. (phamvanphuc90@gmail.com) to obtain the raw data analyzed in this study.

## Abbreviations

anti-NMDAR: anti-N-methyl-D-aspartate receptor
ALT: Alanine aminotransferase
AST: Aspartate aminotransferase
CAP: Community-acquired pneumonia
CAUTI: Catheter-associated urinary tract infection
CLSI: Clinical and Laboratory Standards Institute
CRP: C-reactive protein
CRRT: Continuous renal replacement therapy
CSF: Cerebrospinal fluid
CT scan: Computerized tomography scan
CVC: Central venous catheter
ETA: Endotracheal aspirate
EUCAST: European Committee on Antimicrobial Susceptibility Testing
HAP: Hospital-acquired pneumonia
HSV: Herpes simplex virus
ICU: Intensive Care Unit
INR: International normalized ratio for prothrombin time
IPC: Infection prevention and control
JEV: Japanese encephalitis virus
MALDI-TOF MS: Matrix assisted laser desorption/ionization time-of-flight mass spectrometry
MIC: Minimum inhibitory concentration
MRI: Magnetic resonance imaging
NHTD: National Hospital for Tropical Diseases
RT-PCR: Real-time Polymerase chain reaction
PCT: Procalcitonin
UTI: Urinary tract infections
VAP: Ventilator-associated pneumonia
WBC: White blood cell
